# Supplementary material for: Telomere length in offspring is determined by mitochondrial-nuclear communication at fertilization
Source: Nat Commun. 2025 Mar 14;16:2527. doi: 10.1038/s41467-025-57794-7 (PMC11909127; doi:10.1038/s41467-025-57794-7)
Supplement: Supplementary file 1 — Supplementatary Information [file 41467_2025_57794_MOESM1_ESM.pdf]

SUPPLEMENTARY INFORMATION

Telomere length in offspring is determined by mitochondrial-nuclear communication at fertilization

Yasmyn E. Winstanley[1], Ryan D. Rose[1,2], Alexander P. Sobinoff[3], Linda L. Wu[1], Deepak Adhikari[4], Qing-Hua Zhang[4], Jadon K. Wells[3], Lee H. Wong[5], Hazel H. Szeto[6], Sandra G. Piltz[1,7], Paul Q. Thomas[1,7], Mark A. Febbraio[8], John Carroll[4], Hilda A. Pickett[3], Darryl L. Russell[1], Rebecca L. Robker[1,4*]

[1] Robinson Research Institute, School of Biomedicine, The University of Adelaide, Adelaide, SA, Australia

[2] Genea Fertility SA, St. Andrews Hospital, Adelaide, Australia

[3] Telomere Length Regulation Unit, Children's Medical Research Institute, Faculty of Medicine and Health, University of Sydney, Westmead, NSW, Australia.

[4] Development and Stem Cells Program and Department of Anatomy and Developmental Biology, Monash Biomedicine Discovery Institute, Monash University, Melbourne, Australia

[5] Department of Biochemistry and Molecular Biology, Monash Biomedicine Discovery Institute, Monash University, Melbourne, Australia

[6] Social Profit Network, Menlo Park, CA 94025

[7] South Australian Health & Medical Research Institute, Adelaide, SA, Australia

[8] Monash Institute of Pharmaceutical Sciences, Monash University, Parkville, Victoria, Australia.

* Corresponding author:
Prof Rebecca L. Robker
The University of Adelaide
Adelaide, SA
Australia
Rebecca.robker@adelaide.edu.au
+61 8 8313 8159

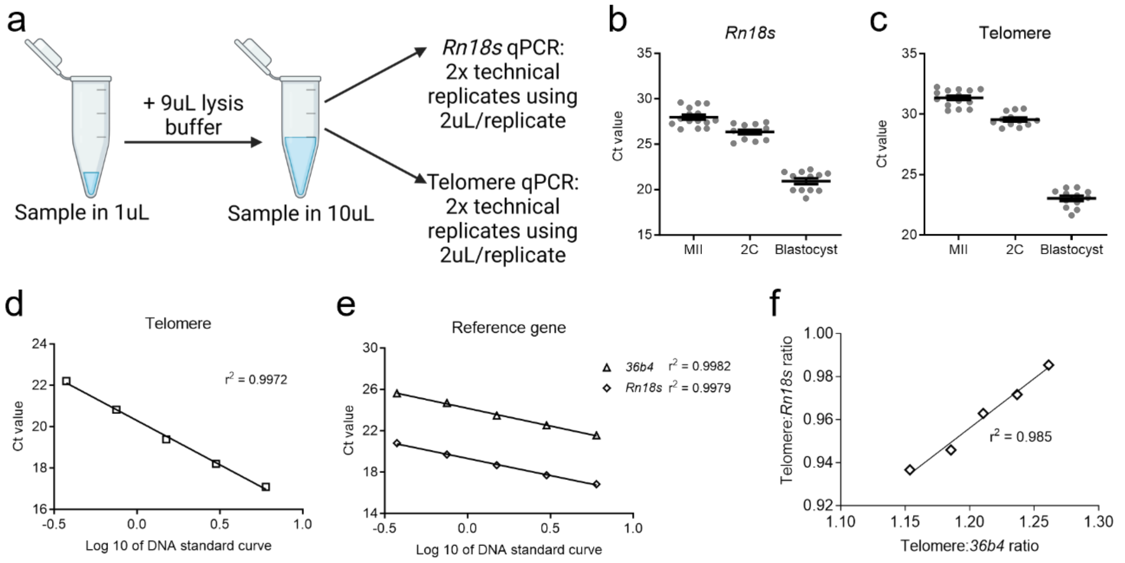

Fig. S1. Telomere length qPCR assay. To enable robust measurement of telomere DNA per cell in individual embryos, a novel qPCR assay was developed. The use of a reference gene (*Rn18s*) enabled normalization of changes in cell number with development. (a) qPCR assay design for quantification of telomere and *Rn18s* sequences. Individual oocytes or embryos are collected in 1µL, and 9µL of lysis solution added and used for telomere and *Rn18s* analysis. The sensitivity of the assay was validated using MII oocytes (n=15), 2-cell (2C) and blastocyst stage (n=12) embryos; with the expected decrease in Ct values with increasing cell number demonstrated for *Rn18s* (b), and telomere (c). Specifically, a doubling in cell number from 1 (MII oocyte) to 2 (2-cell embryo) was associated with a 1 cycle decrease in Ct value for *Rn18s*, while a blastocyst was associated with a 6 cycle lower Ct value than a 2-cell. Efficiency of primers and linearity of sequence amplification with input DNA concentration was validated (d, e). DNA was extracted from one mouse ovary and serially diluted (1:2) to 6ng/µL, 3ng/µL, 1.5ng/µL, 0.75ng/µL, and 0.375ng/µL which were used to demonstrate efficiency of the telomere primers (d), and *Rn18s* primers, with *36b4* as a comparator (e) under identical cycling conditions. To test for linearity, DNA concentration was log10 transformed for plotting with Ct values, with r^2 values shown. Each data point is derived from triplicate PCR reactions. Each primer pair showed a high degree of linearity, with correlation coefficients consistently above 0.995 (d, e). Comparison of multi-copy reference gene *Rn18s* to the single-copy gene *36b4*, showed the Ct of detection decreased by a value of 1 with each halving of input DNA concentration for both *Rn18s* and *36b4* primers, and correlation coefficients were above 0.995 for both primer sets (e), demonstrating the suitability of *Rn18s* as a reference gene. The telomere:reference gene ratio for *36b4* and *Rn18s* was calculated and linear correlation analysis performed (f) demonstrating the suitability of both methods for analysis of samples where DNA amount is not limiting (i.e. fetal tissues). Individual data points plotted, horizontal lines are mean±SEM (b,c). Source data are provided as a Source Data file. (a) Created in BioRender. Gordon, Y. (2025) https://BioRender.com/b48p191.

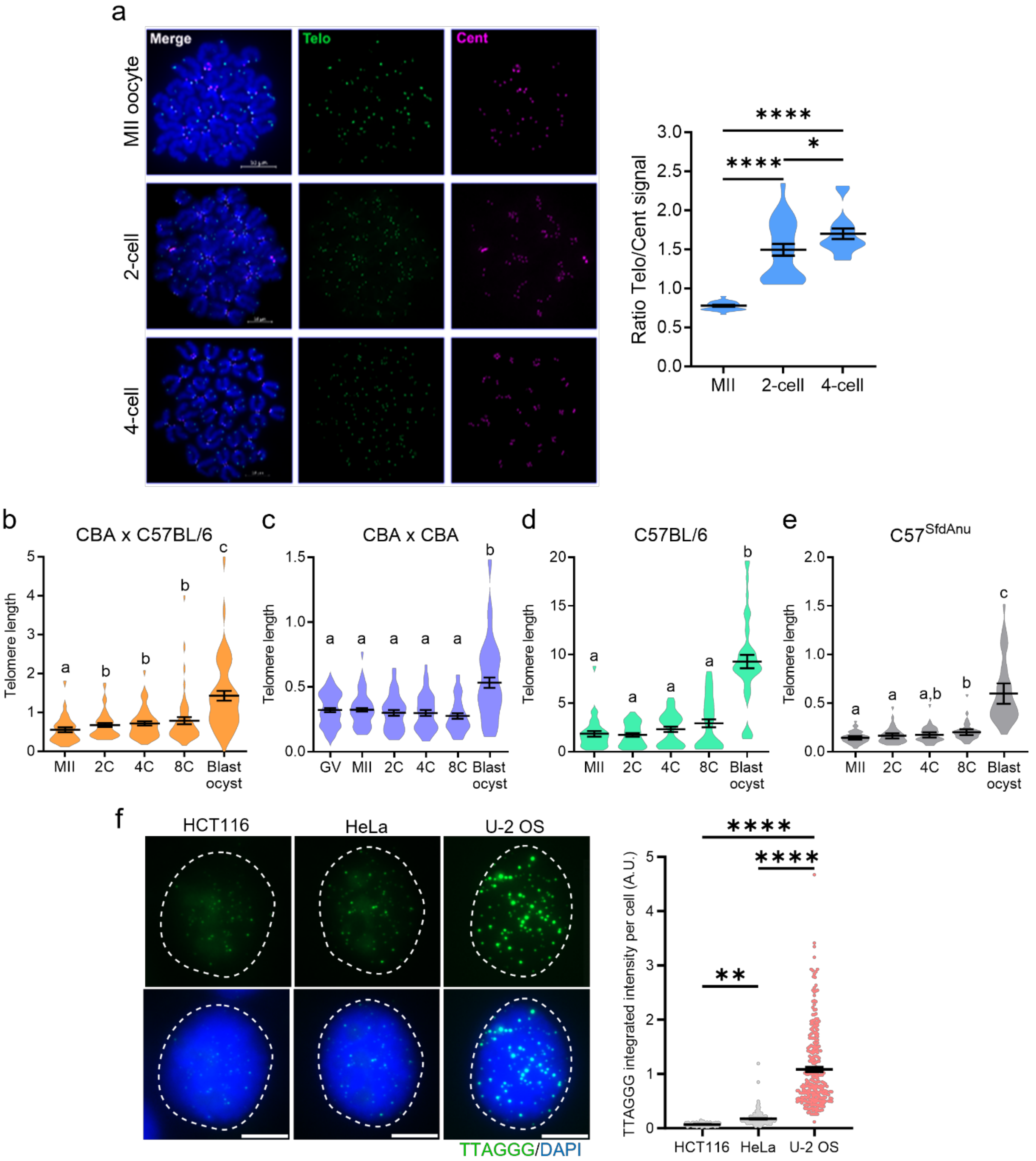

Fig. S2. Telomeres elongate within the first cell cycle. (a) qFISH for telomere (green) and centromere (magenta) with DNA stain (blue) in representative MII oocyte, 2C and 4C embryo cell (a, left). Telomere to centromere fluorescence signal ratio indicates relative telomere length in C57BL/6 x CBA.F1 IVF embryos (a, right; n=24 MII, n=25 2-cell, n=16 4-cell stage cells). Telomere length ($2^{-\Delta\Delta Ct}$) in individual oocytes and embryos from different strains of mice: CBA x C57BL/6 (b; n=31 MII, n=51 2C, n=50 4C, n=52 8C, n=51 blastocyst), CBA x CBA (c; n=67 GV, n=94 MII, n=37 2C, n=38 4C, n=39 8C, n=52 blastocyst), C57BL/6 x C57BL/6 (d; n=32 MII, n=39 2C, n=37 4C, n=42 8C, n=41 blastocyst), or C57BL/6JSfdAnu-Alms1($C57^{SfdAnu}$) x $C57^{SfdAnu}$ (e; n=42 MII, n=40 2C, n=38 4C, n=38 8C, n=38 blastocyst). Violin plots show population distribution, and horizontal

lines are mean ± SEM. (f) Standards for telomere FISH showing telomere (green) and DAPI DNA stain (blue) in cell lines known to have homogeneous short telomere lengths (HCT116, HeLA; average 4-5kb) or long heterogeneous telomeres (U-2 OS; average ~36kb) (f, left), and relative telomere length per cell quantified (f, right; n=282 nuclei per group). Individual data points are plotted, horizontal lines are mean ± SEM. Data analyzed using one-way ANOVA (a) or linear mixed-effects model (panels b-e), for exact P values see Supplementary Table 10. Source data are provided as a Source Data file.

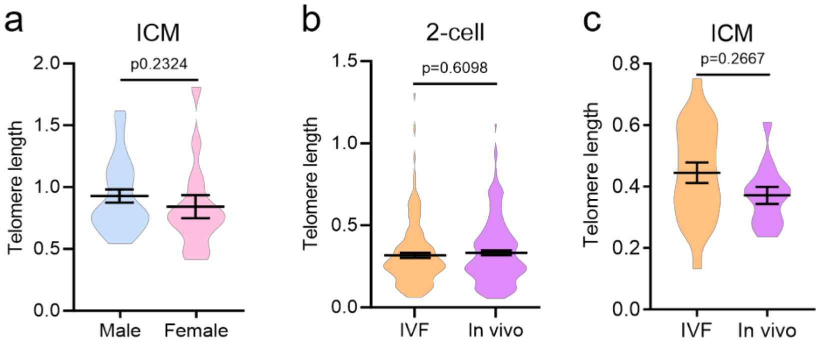

Fig. S3. Telomere length in male and female embryos generated by IVF (*in vitro* fertilization) or *in vivo* (mating). ICM telomere length ($2^{-\Delta\Delta Ct}$) in male (n=30) and female (n=15) embryos (a) generated by IVF. Telomere length ($2^{-\Delta\Delta Ct}$) in IVF-derived compared to *in vivo*-derived 2-cell embryos (b; n=157 IVF and n=176 *in vivo*) and ICMs (c; n=22 IVF and n=13 *in vivo*). Violin plots represent the population distributions, and horizontal lines are mean ± SEM. Data was log transformed for statistical analysis using a two-sided unpaired t-test, with exact P values shown. Source data are provided as a Source Data file.

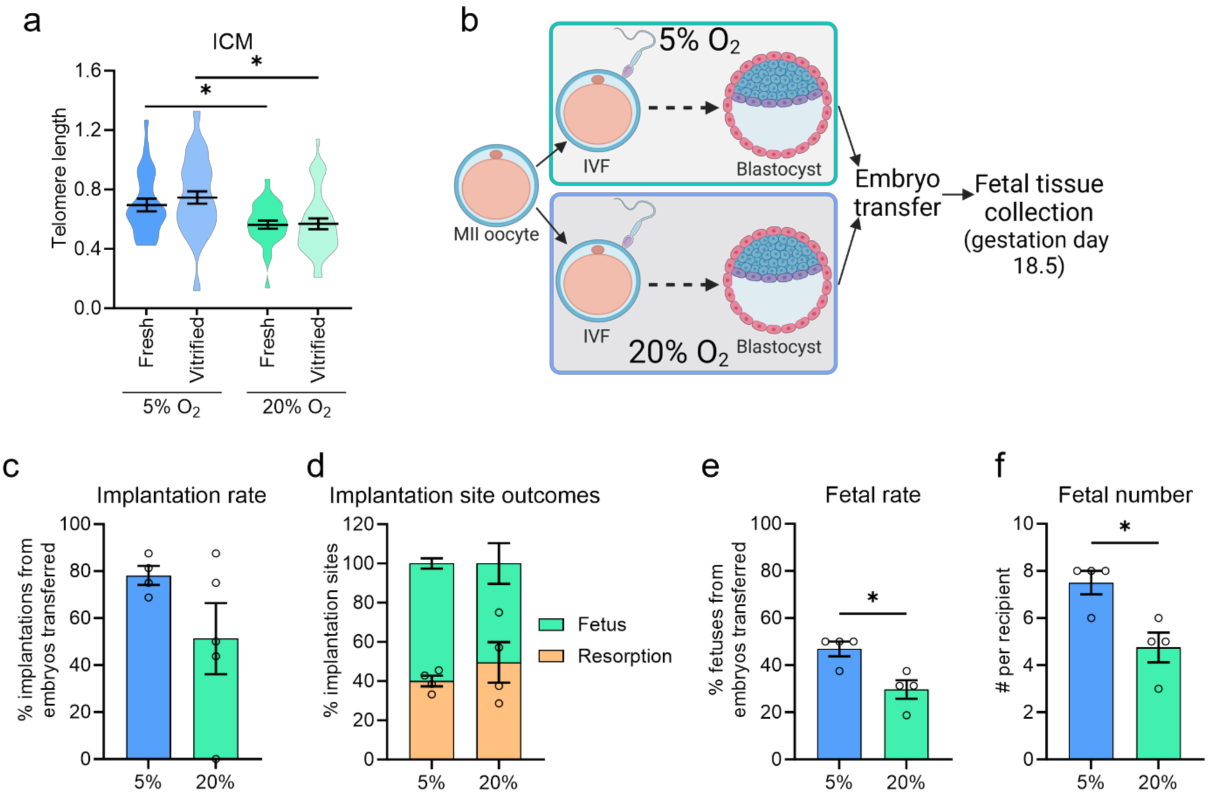

Fig. S4. High oxygen culture impairs fetal development following embryo transfer. (a) Telomere length ($2^{-\Delta\Delta Ct}$) was assessed in ICMs derived from blastocysts that were 'fresh' (i.e. continuous culture from fertilization) or vitrified at the morula stage and then thawed before culture to the blastocyst stage, to confirm that vitrification did not impact embryo telomere elongation (a; n=25 fresh and n=42 vitrified embryos at 5% O_2, and n=28 fresh and n=36 vitrified embryos at 20% O_2). qPCR data was log transformed for statistical analysis using one-way ANOVA. Shaded areas represent the population distributions, and horizontal lines are mean ± SEM (a). Blastocyst stage embryos were transferred to pseudo-pregnant recipient females (n=4 females for 5% O_2 and n=5 females for 20% O_2 embryos) at day 2.5 of pregnancy and fetuses collected at day 18.5 (b). The number of uterine sites where an embryo had implanted was counted and expressed as a percentage of the embryos transferred to give implantation rate (c). Whether the implantation resulted in a fetus or resorption was noted (d; data points for % resorptions shown). The proportion of resulting fetuses from total embryos transferred was calculated (e), as well as the number of fetuses per recipient (f). Individual data points are plotted, horizontal lines are mean ± SEM (c, e-f), or ratios ± SEM (d). Embryo transfer and fetal outcomes and fetal tissue length were assessed using a two-sided unpaired t-test, for exact P values see Supplementary Table 11. Source data are provided as a Source Data file. (b) Created in BioRender. Gordon, Y. (2025) https://BioRender.com/t45y682.

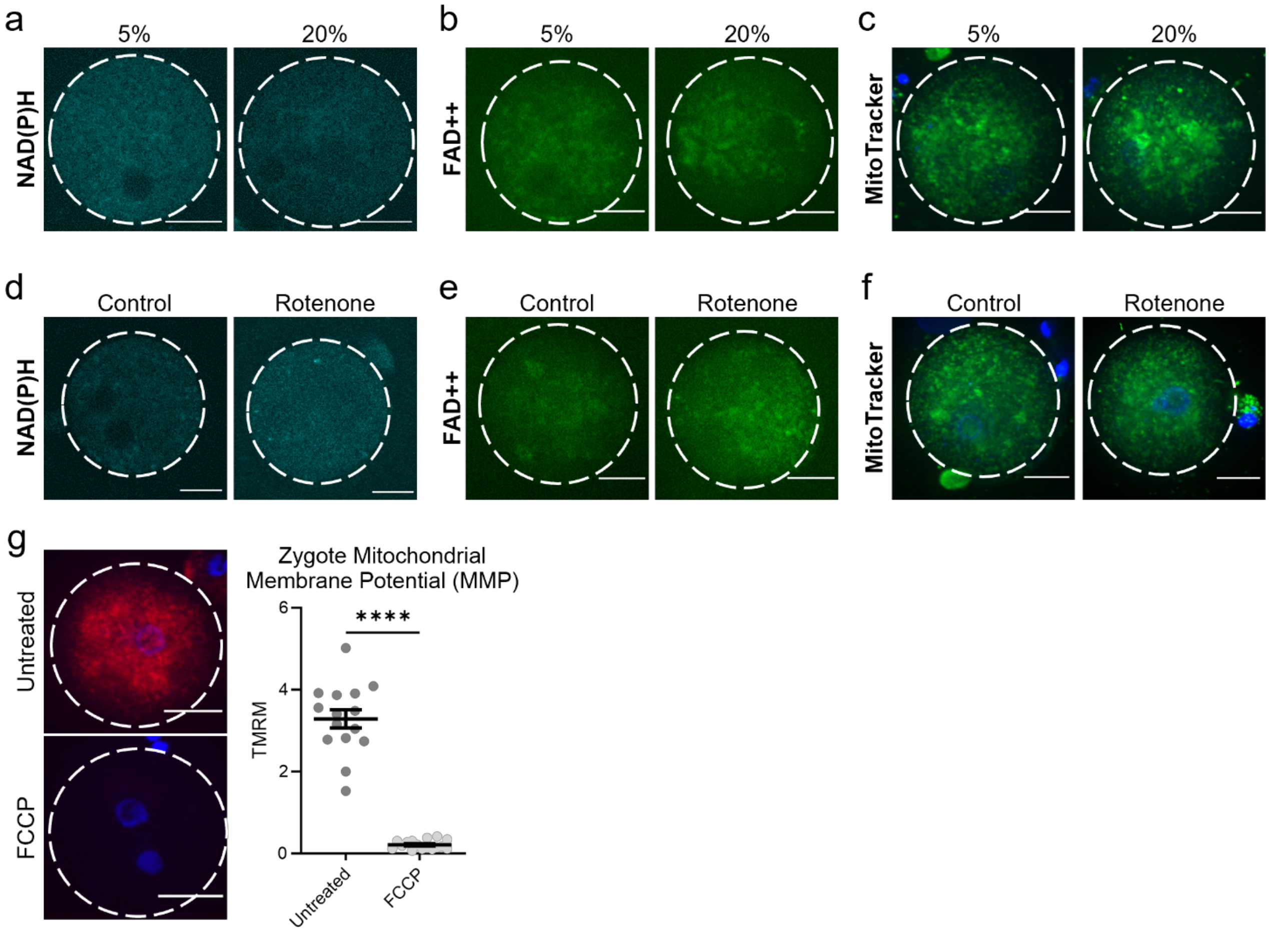

Fig. S5. Tricarboxylic acid (TCA) cycle metabolites and mitochondrial content in high oxygen (20%) and rotenone stress models. Representative images of zygotes for quantitation of levels of NAD(P)H (blue, a, d) and FAD++ (green, b, e) 6h post-IVF in high oxygen (20%) and rotenone models, respectively. Zygote mitochondrial content was assessed using MitoTracker Green (green) in high oxygen (20%, c) and rotenone (f) models. To demonstrate the specificity of TMRM (red) as an indicator of MMP, after TMRM incubation (with Hoechst-3342 DNA stain (blue)), zygotes were exposed to 1μM FCCP for 10 minutes prior to imaging (g). FCCP-mediated uncoupling abrogated the TMRM/MMP signal, analyzed via two-sided unpaired t-test, $p<0.0001$. Individual data points are plotted, horizontal lines are mean ± SEM**.** Source data are provided as a Source Data file.

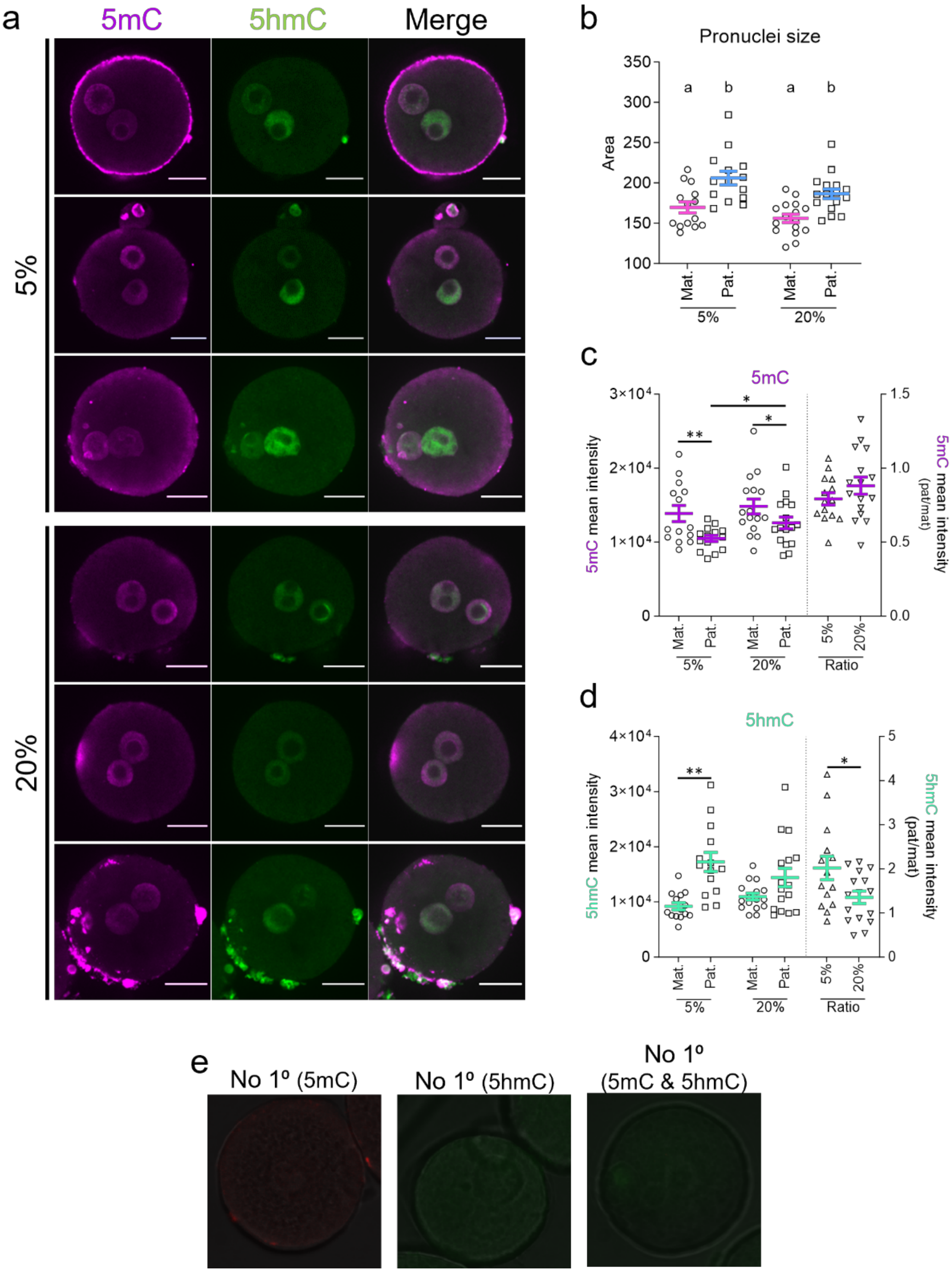

Fig. S6. High oxygen culture alters zygotic epigenetic reprogramming. Zygotes were collected 10 hours after fertilization and immuno-labelled with anti-5-methylcytosine (5mC; magenta) and anti-5-hydroxymethylcytosine (5hmC; green) antibodies. Representative images are shown (a). Pronuclei size was measured (b) and fluorescent signal intensity levels were quantified for 5mC (c) and 5hmC (d) in maternal and paternal pronuclei (n=14 5% O_2 and 16 20% O_2 zygotes). Data presented as signal intensity for maternal (Mat.) and paternal (Pat.) pronuclei on the left axis, and the ratio of the fluorescence signal in paternal versus maternal pronuclei of the same zygote on the right axis. Data analyzed using two-sided paired t-test for comparisons between maternal and paternal

pronuclei of the same zygote, and two-sided unpaired t-test for pronuclei comparisons and ratio comparisons between groups, for exact P values see Supplementary Table 12. Individual data points are plotted, horizontal lines are mean ± SEM**.** Secondary only controls for 5mC (left), 5hmC (middle), and 5mC and 5hmC (right) in which the primary antibody was omitted to validate specificity (e). Source data are provided as a Source Data file.

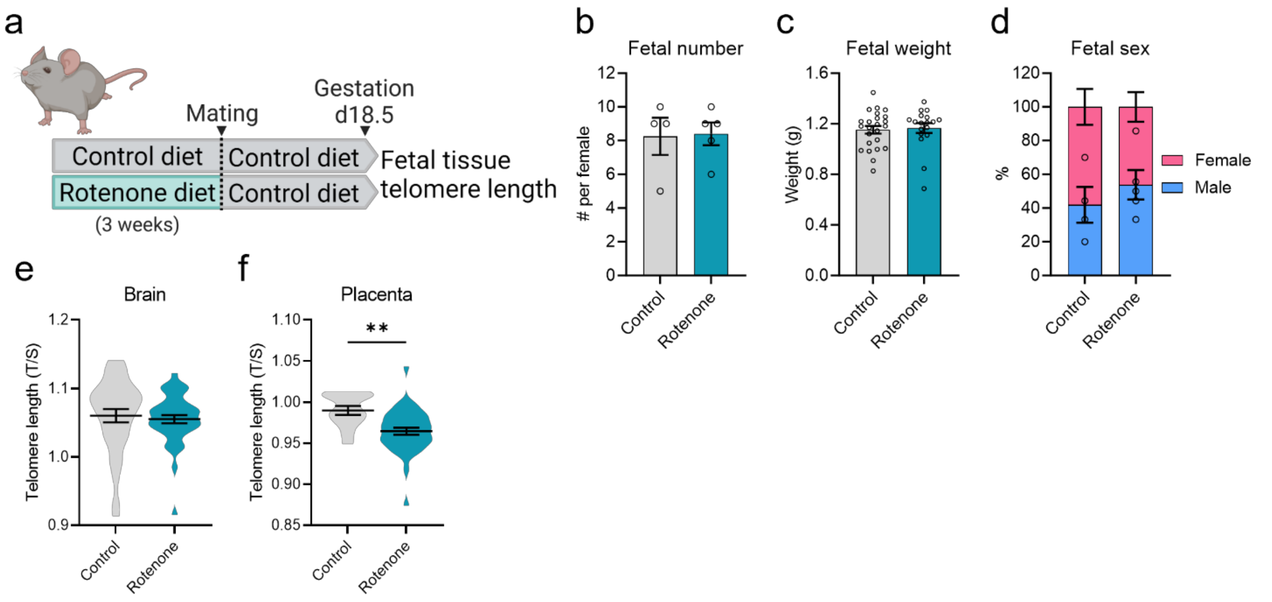

Fig. S7. Rotenone exposure prior to natural conception reduces placenta telomere length. A cohort of female mice that had consumed either control or rotenone diet for three weeks were paired (1:1) with a male, and upon presence of a vaginal copulatory plug, were transferred to control diet to limit gestational rotenone exposure. Fetuses were collected on day 18.5 of pregnancy for analysis (a). Fetal number per female (b; n=4 control and n=5 rotenone females), fetal weight (c; n=24 control and n=18 rotenone fetuses) and fetal sex (d; n=4 control and n=5 rotenone litters; data points for % males shown) were determined. Telomere length (telomere (T)/ *Rn18s* (S)) in fetal brains (e; n=32 control and n=39 rotenone fetuses) and placentas (f; n=14 control and n=39 rotenone placentas) were analyzed via qPCR. Data analyzed using two-sided unpaired t-test, for exact P values see Supplementary Table 13. Individual data points are plotted, horizontal lines are mean ± SEM (b, c), or ratios ± SEM (d). Violin plots represent the population distributions, and horizontal lines are mean ± SEM (e, f). Source data are provided as a Source Data file. (a) Created in BioRender. Gordon, Y. (2025) https://BioRender.com/b05w439.

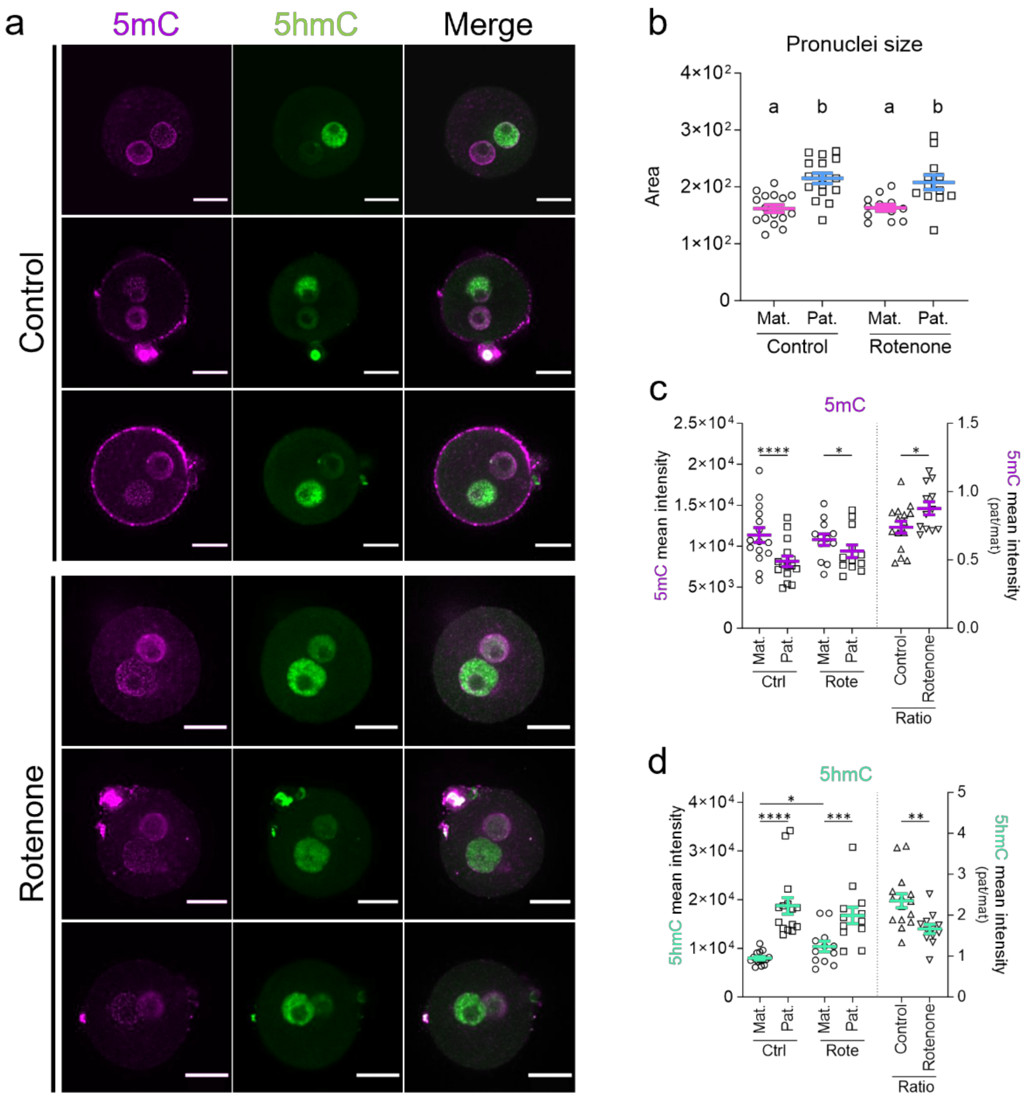

Fig. S8. Maternal rotenone exposure alters zygotic epigenetic reprogramming. Zygotes were collected 10 hours after fertilization and immuno-labelled with anti-5-methylcytosine (5mC; magenta) and anti-5-hydroxymethylcytosine (5hmC; green) antibodies (a). Pronuclei size was measured (b) and fluorescent signal intensity levels were quantified for 5mC (c) and 5hmC (d) in maternal and paternal pronuclei (n=15 control (Ctrl) and n=12 rotenone (Rote) zygotes per group). Data presented as signal intensity for maternal (Mat.) and paternal (Pat.) pronuclei on the left axis, and the ratio of the fluorescence signal in paternal versus maternal pronuclei of the same zygote on the right axis. Data analyzed using two-sided paired t-test for comparisons between maternal and paternal pronuclei of the same zygote, and two-sided unpaired t-test for pronuclei comparisons and ratio comparisons between groups, for exact P values see Supplementary Table 14. Individual data points are plotted, horizontal lines are mean ± SEM. Source data are provided as a Source Data file.

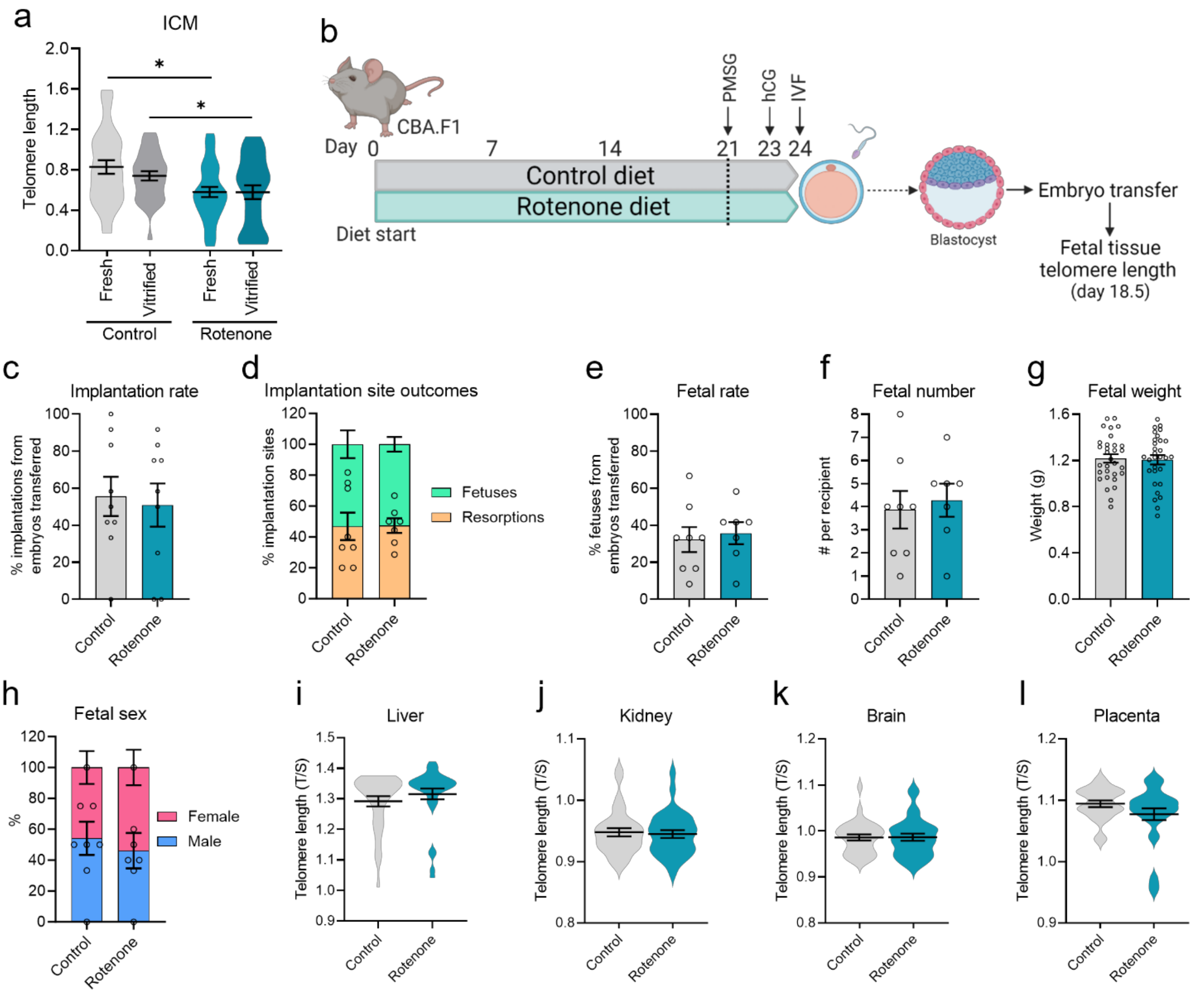

Fig. S9. Oocyte rotenone exposure does not affect fetal outcomes following embryo transfer. (a) Telomere length was assessed in ICMs derived from blastocysts that were 'fresh' (i.e. continuous culture from fertilization) or vitrified at the morula stage and then thawed before culture to the blastocyst stage, to confirm that vitrification did not impact embryo telomere elongation (a; n=31 fresh and n=28 vitrified control embryos, and n=34 fresh and n=27 vitrified rotenone-exposed embryos). ICM qPCR data was log transformed for statistical analysis using one-way ANOVA. Blastocysts were transferred to pseudo-pregnant recipient females (n=9 females for each embryo type) at day 2.5 of pregnancy and fetuses were collected at day 18.5 for analysis (b). The number of uterine sites where an embryo had implanted was counted and expressed as a percentage of the embryos transferred to give implantation rate (c). Whether the implantation resulted in a fetus or resorption was noted (d; data points for % resorptions shown). The proportion of resulting fetuses from total embryos transferred was calculated (e), as well as the number of fetuses per recipient (f). n=8 control and n=7 rotenone litters (d-f). Fetal characteristics (n=31 from control and n=30 from rotenone-exposed embryos) including weight (g) and fetal sex (h; fetuses from n=8 control and n=7

rotenone litters; data points for % males shown) were analyzed. Telomere length in fetal livers (i), kidney (j), brain (k) (n=31 from control and n=30 from rotenone-exposed fetuses), and placenta (l; n=23 control and n=26 rotenone placentas) were analyzed via qPCR. Fetal outcomes (c-h), and fetal liver, kidney, brain and placenta telomere length (i-l), were assessed using two-sided unpaired t-test. For exact P values see Supplementary Table 15. Violin plots represent the population distributions, and horizontal lines are mean ± SEM (a, i-l). Individual data points are plotted, horizontal lines are mean ± SEM (c, e-g), or ratios ± SEM (d, h). Source data are provided as a Source Data file. (b) Created in BioRender. Gordon, Y. (2025) https://BioRender.com/f30t440.

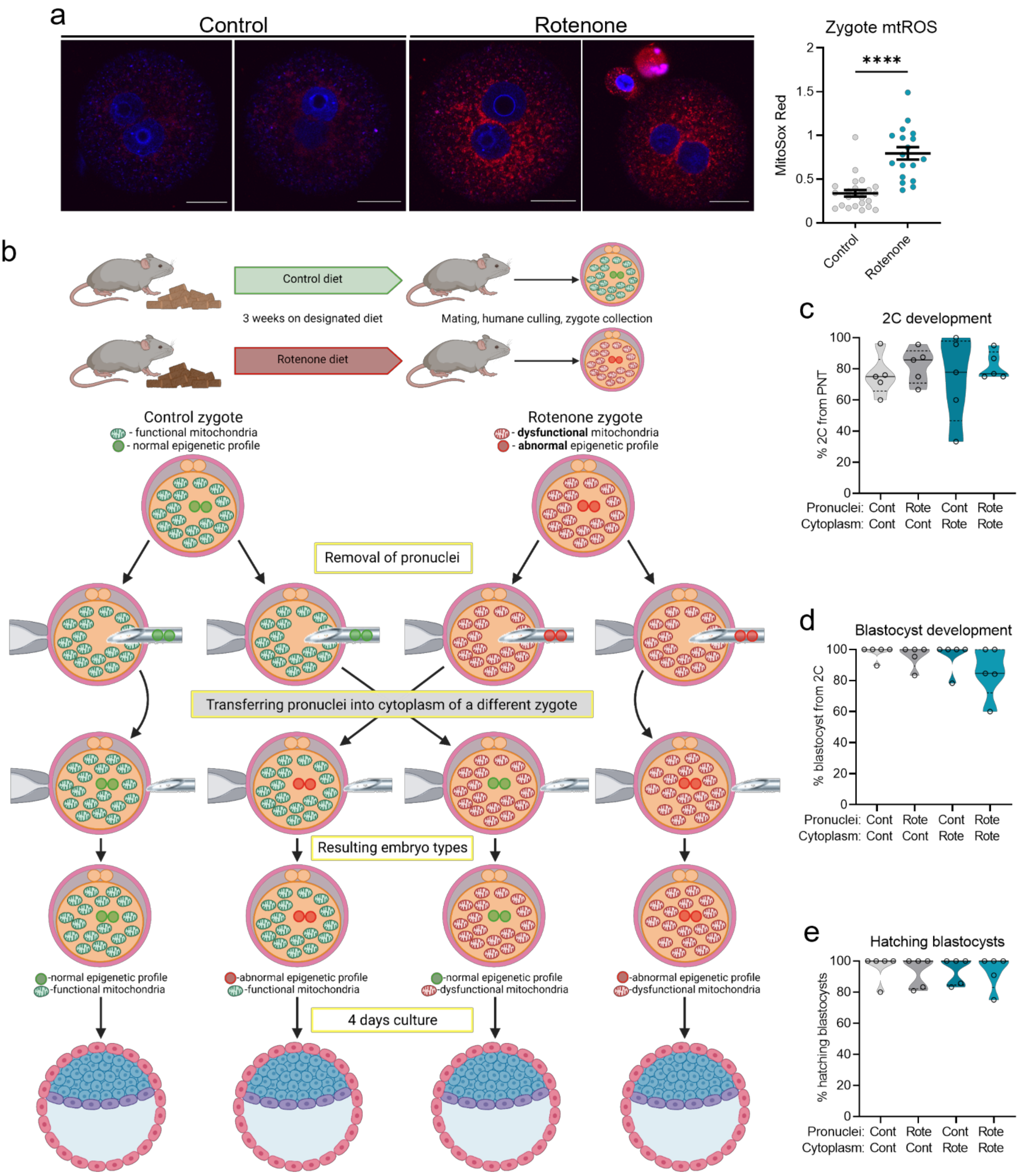

Fig. S10. Pronuclei transfer between control and rotenone-exposed zygotes. *In vivo* fertilized zygotes were collected at 20h post-hCG (~8 hours post-fertilization), and labelled with MitoSOX Red (MSR) mtROS indicator (and DNA stain Hoechst-3342; blue) (a) and red fluorescence (CTCF) was determined (a; n=25 control and n=18 rotenone). Individual data points are plotted, horizontal lines are mean ± SEM, data analyzed via unpaired t-test, **** p<0.0001. Schematic representation of pronuclear transfer between zygotes and subsequent embryo culture (b). Zygotes (18h post-hCG) were obtained from female mice that had consumed control (Cont) or rotenone (Rote) -containing

(150ppm) diet for 3 weeks prior to hormone stimulation and mating; and cultured for 2h. Zygotic pronuclei and cytoplasm were recombined to produce 4 embryo types derived either from control or rotenone-exposed zygotes only (pronuclei were transferred between zygotes from the same group) or a combination of pronuclei and cytoplasm derived from control and rotenone-exposed zygotes. Embryo development after pronuclear transfer was assessed at the 2-cell (c) and blastocyst (d) stages, as well as proportion of hatching blastocysts (e). Development data plotted as violin plots with median (solid line) and quartiles (dashed lines) shown, and analyzed using one-way ANOVA. Embryos were generated from 5 independent rounds of pronuclei transfers where reconstructed zygotes were produced for all four groups in each round, generating a minimum of 7 reconstructed zygotes for each group in each round. For exact P values see Supplementary Table 16. Source data are provided as a Source Data file. (b) Created in BioRender. Gordon, Y. (2025) https://BioRender.com/u90a181.

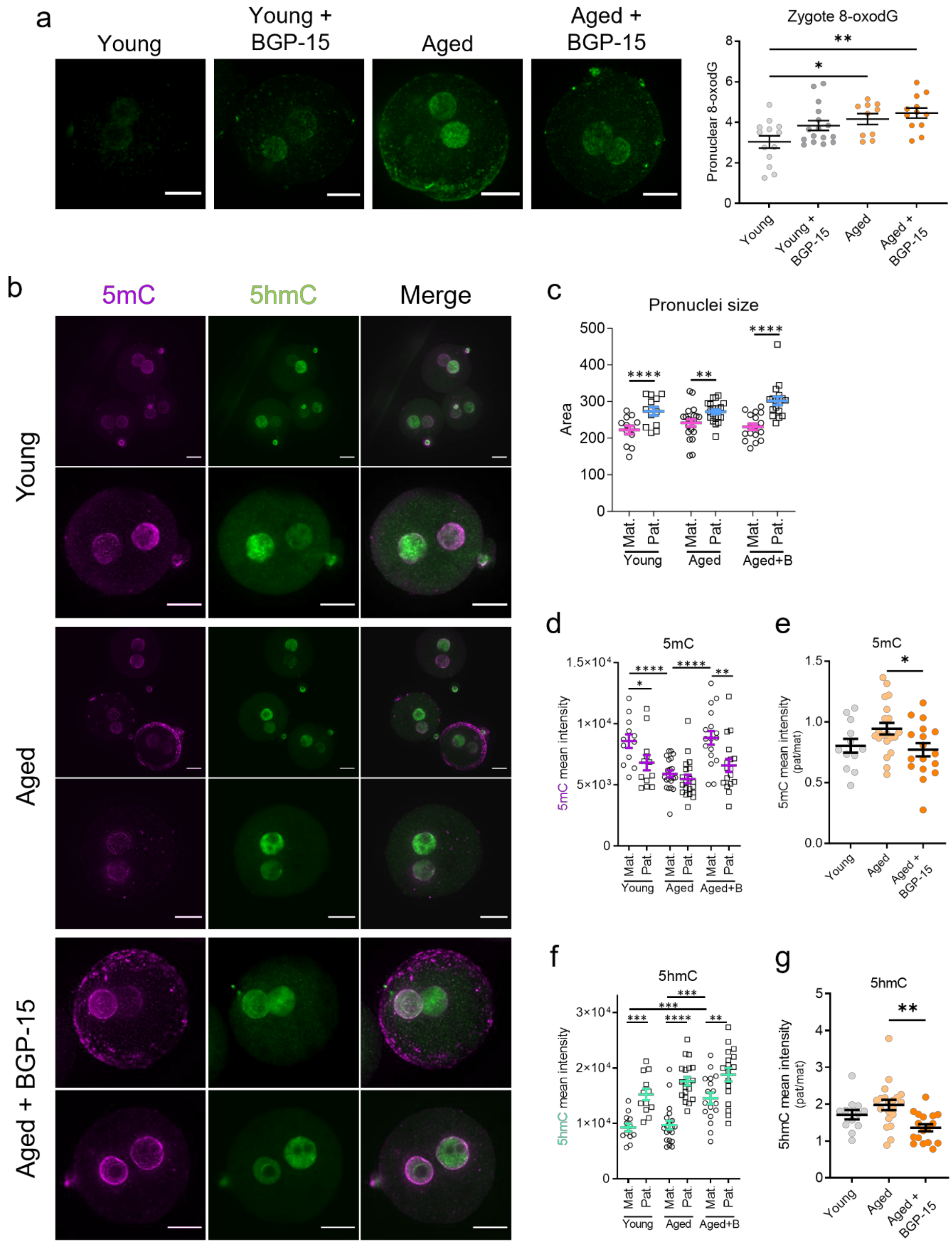

Fig. S11. Advanced maternal age alters zygotic epigenetic reprogramming that is modified by BGP-15 treatment. Zygotes (8h post-IVF) from young, aged, and aged females treated with BGP-15 (+BGP-15) were immuno-labeled with anti-8-oxodG (green) and fluorescence intensity in the pronuclei quantified (a; n=13 young, n=16 young + BGP-15, n=10 aged and n=12 aged + BGP-15 zygotes). Zygotes from aged females had significantly increased levels of pronuclear 8-oxodG. Zygotes were collected 10 hours after *in vitro* fertilization and immuno-labelled with anti-5-

methylcytosine (5mC; magenta) and anti-5-hydroxymethylcytosine (5hmC; green) antibodies (b). Pronuclei size was measured (c) and fluorescent signal intensity levels were quantified for 5mC (d) and 5hmC (f) in maternal and paternal pronuclei (n=12 young, n=20 aged and n=17 aged + BGP-15 (+B) zygotes), and the ratio of the fluorescence signal in paternal (Pat.) versus maternal (Mat.) pronuclei of the same zygote (e, g). Zygotes from aged females exhibited a trend towards altered relative levels of 5mC and 5hmC in pronuclei, that were not identical to the changes induced by the other oxidative stress models. Interestingly however, following treatment with BGP-15, this nuclear patterning was similar to that of zygotes from young mice, indicating that BGP-15 treatment either directly or via modulation of mtROS, influences nuclear DNA. Individual data points are plotted, horizontal lines are mean ± SEM. Data analyzed using two-sided paired t-test for comparisons between maternal and paternal pronuclei of the same zygote (c, d, f), and one-way ANOVA for pronuclei comparisons (including ratio comparisons) between groups, for exact P values see Supplementary Table 17. Source data are provided as a Source Data file.

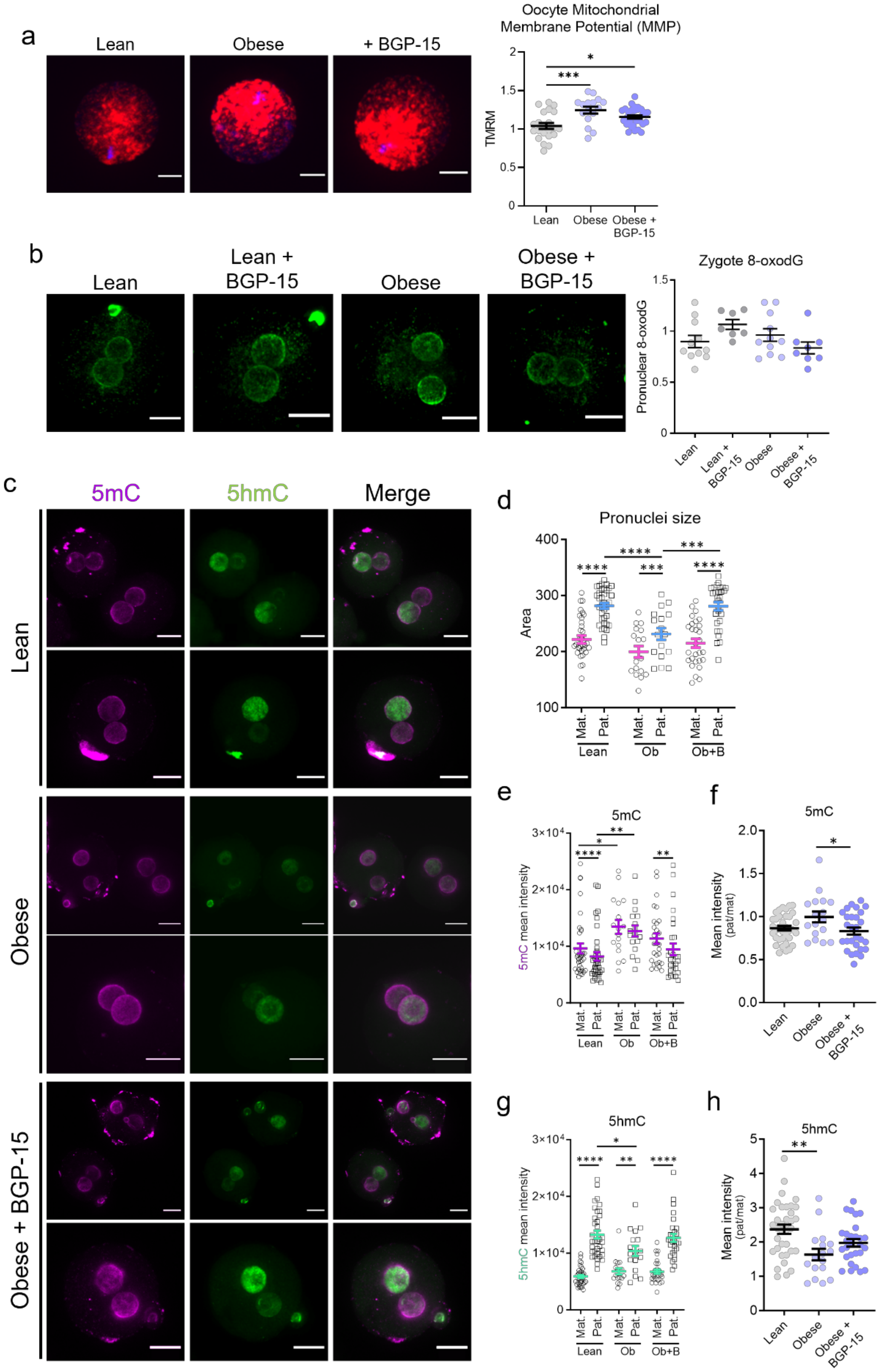

Fig. S12. Maternal obesity results in alterations to zygotic epigenetic reprogramming that are reversed by BGP-15 treatment. Oocytes from lean, obese, and obese females treated with BGP-15 were stained with TMRM (red) and Hoechst-3342 (blue) and red fluorescence determined (a; n=22 lean, n=16 obese, n=32 obese+BGP-15). Zygotes (8h post-IVF) from lean and obese (Ob) females

with or without BGP-15 treatment (+BGP-15 or +B) were immuno-labeled with anti-8-oxodG and fluorescence intensity in the pronuclei quantified (b; n=11 lean, n=7 lean+ BGP-15, n=11 obese and n=8 obese + BGP-15). Data analyzed using one-way ANOVA (a, b). Zygotes were collected 10 hours after *in vitro* fertilization and immuno-labelled with anti-5-methylcytosine (5mC; magenta) and anti-5-hydroxymethylcytosine (5hmC; green) antibodies (c). Pronuclei size was measured (d) and fluorescent signal intensity levels were quantified for 5mC (e) and 5hmC (g) in maternal and paternal pronuclei, and the ratio of the fluorescence signal in paternal (Pat.) versus maternal (Mat.) pronuclei of the same zygote (f, h) (d-h; n=35 lean, n=17 obese, n=28 obese+BGP-15). Individual data points are plotted, horizontal lines are mean ± SEM. Data analyzed using two-sided paired t-test for comparisons between maternal and paternal pronuclei of the same zygote, and one-way ANOVA for pronuclei comparisons (including ratio comparisons) between groups, for exact P values see Supplementary Table 18. Source data are provided as a Source Data file.

Supplementary Table 1. Statistical analysis of data presented in Figure 1.

Fig.	Parameter	Groups	a,b	a,c	a,d	b,c	b,d	c,d	a,e; b,e; c,e	d,e
**1b**	Telomere length	**a:**MII **b:**2C **c:**4C **d:**8C **e:**Blast	0.043 *	0.008 ***	<0.0001 ****	0.572 NS	0.009 **	0.031 *	<0.0001 ****	0.015 *
**1c**	Telomere length	**a:**TE **b:**ICM	0.0143 *	n/a	n/a	n/a	n/a	n/a	n/a	n/a
**1e**	Telomere length	**a**:D5 **b:**D6	0.2365 NS	n/a	n/a	n/a	n/a	n/a	n/a	n/a
**1g**	Telomere length	**a:**5% **b:**20%	0.1273 NS	n/a	n/a	n/a	n/a	n/a	n/a	n/a
**1h**	Telomere length	**a:**5%mor **b:**5%ICM **c:**20%mor **d:**20%ICM	0.0004 ***	0.0178 *	0.8385 NS	<0.0001 ****	0.0178 *	0.0004 ***	n/a	n/a
**1i**	Telomere length	**a:**5% **b:**20%	<0.0001 ****	n/a	n/a	n/a	n/a	n/a	n/a	n/a
**1j**	TTAGGG intensity	**a:**5% **b:**20%	<0.0001 ****	n/a	n/a	n/a	n/a	n/a	n/a	n/a

Supplementary Table 2. Statistical analysis of data presented in Figure 2.

Fig.	Parameter	Groups	a,b	a,c	a,d; b,d; d,f; e,g	a,e; a,f; a,g; b,e; b,f; b,g; c,e; c,g; d,e; d,g; e,f; e,h; f,g; f,h; g,h	a,h; b,h; c,d; d,h	b,c	c,f	c,h
**2b**	MitoSox Red	**a:** 5% **b:** 20%	<0.0001 ****	n/a	n/a	n/a	n/a	n/a	n/a	n/a
**2c**	NAD(P)H	**a:** 5% **b:** 20%	<0.0001 ****	n/a	n/a	n/a	n/a	n/a	n/a	n/a
**2d**	FAD++	**a:** 5% **b:** 20%	0.6452 NS	n/a	n/a	n/a	n/a	n/a	n/a	n/a
**2e**	Mitotracker	**a:** 5% **b:** 20%	0.1888 NS	n/a	n/a	n/a	n/a	n/a	n/a	n/a
**2f**	TMRM	**a:** 5%zyg **b:** 20%zyg **c:** 5%8C **d:** 20%8C **e:** 5%mor **f:** 20%mor **g:** 5%blast **h:** 20%blast	>0.999 NS	0.0558 NS	>0.5 NS	<0.0001 ****	>0.9 NS	0.0343 *	0.0604 NS	0.6985 NS
**2g**	8-oxodG	**a:** 5% **b:** 20%	0.0056 **	n/a	n/a	n/a	n/a	n/a	n/a	n/a
**2h**	5mC	**a:** 5% **b:** 20%	0.2283 NS	n/a	n/a	n/a	n/a	n/a	n/a	n/a
**2h**	5hmC	**a:** 5% **b:** 20%	0.0292 *	n/a	n/a	n/a	n/a	n/a	n/a	n/a

Supplementary Table 3. Statistical analysis of data presented in Figure 3.

Fig.	Parameter	Groups	a,b
**3b**	Telomere length	**a:**control<br>**b:**rotenone	0.2268<br>NS
**3c**	Telomere length	**a:**control<br>**b:**rotenone	0.0016<br>**
**3d**	Telomere length	**a:**control<br>**b:**rotenone	0.0206<br>*

Supplementary Table 4. Statistical analysis of data presented in Figure 4.

Fig.	Parameter	Groups	a,b	a,c; b,d	a,d; b,c	c,d	e,f	a,e; c,e; b,f; d,f
**4b**	MitoSox Red	**a:**control<br>**b:**rotenone	<0.0001<br>****	n/a	n/a	n/a	n/a	n/a
**4c**	NAD(P)H	**a:**control<br>**b:**rotenone	0.0049<br>**	n/a	n/a	n/a	n/a	n/a
**4d**	FAD++	**a:**control<br>**b:**rotenone	0.009<br>**	n/a	n/a	n/a	n/a	n/a
**4e**	Mitotracker	**a:**control<br>**b:**rotenone	0.1673<br>NS	n/a	n/a	n/a	n/a	n/a
**4f**	8-oxodG	**a:**control<br>**b:**rotenone	0.0367<br>*	n/a	n/a	n/a	n/a	n/a
**4g**	5mC	**a:**control<br>**b:**rotenone	0.0441<br>*	n/a	n/a	n/a	n/a	n/a
**4g**	5hmC	**a:**control<br>**b:**rotenone	0.0038<br>**	n/a	n/a	n/a	n/a	n/a
**4h**	TMRM	**a:**control zyg<br>**b:**rotenone zyg<br>**c:**control 8C<br>**d:**rotenone 8C	<0.0001<br>****	<0.0001<br>****	<0.0001<br>****	0.0107<br>*	n/a	n/a
**4i**	Telomere length	**a:**control MII<br>**b:**rotenone MII<br>**c:**control 8-cell<br>**d:**rotenone 8-cell<br>**e:**control blastocyst<br>**f:**rotenone blastocyst	0.0430<br>*	<0.0001<br>****	n/a	0.0160<br>*	0.0297<br>*	<0.0001<br>****
**4j**	Telomere length	**a:**control<br>**b:**rotenone	0.0075<br>**	n/a	n/a	n/a	n/a	n/a
**4k**	TTAGGG intensity	**a:**control<br>**b:**rotenone	<0.0001<br>****	n/a	n/a	n/a	n/a	n/a
**4l**	Telomere length	**a:**control<br>**b:**rotenone	0.0363<br>*	n/a	n/a	n/a	n/a	n/a

Supplementary Table 5. Statistical analysis of data presented in Figure 5.

Fig.	Parameter	Groups	a,b	a,c	a,d	b,c	b,d	c,d
**5b**	MitoSox Red	**a:**control<br>**b:**cont+BGP-15<br>**c:**rotenone<br>**d:**rote+B	0.7541<br>NS	0.0006<br>***	0.9795<br>NS	<0.0001<br>****	0.2010<br>NS	0.0026<br>**
**5c**	Telomere length	**a:**control<br>**b:**cont+B<br>**c:**rotenone<br>**d:**rote+B	0.1766<br>NS	0.0240<br>*	0.9822<br>NS	0.8004<br>NS	0.2361<br>NS	0.0296<br>*
**5e**	MitoSox Red	**a:**control<br>**b:**cont+B<br>**c:**rotenone<br>**d:**rote+B	0.9789<br>NS	<0.0001<br>****	0.3323<br>NS	<0.0001<br>****	0.8505<br>NS	<0.0001<br>****
**5f**	Telomere length	**a:**control<br>**b:**cont+B<br>**c:**rotenone<br>**d:**rote+B	0.2722<br>NS	0.0021<br>**	0.8347<br>NS	0.2176<br>NS	0.6715<br>NS	0.0099<br>**

Supplementary Table 6. Statistical analysis of data presented in Figure 6.

Fig.	Parameter	Groups	a,b	a,c	a,d	b,c	b,d	c,d
**6b**	MitoSox Red	**a:**control<br>**b:**cont+BGP-15<br>**c:**rotenone<br>**d:**rote+BGP-15	<0.0001<br>****	0.9923<br>NS	n/a	<0.0001<br>****	n/a	n/a
**6c**	Telomere length	**a:**control<br>**b:**cont+BGP-15<br>**c:**rotenone<br>**d:**rote+BGP-15	0.9577<br>NS	0.0204<br>*	>0.9999<br>NS	0.007<br>**	0.9578<br>NS	0.0267<br>*
**6d**	MitoSox Red	**a:**control<br>**b:**cont+MitoQ<br>**c:**rotenone<br>**d:**rote+MitoQ	0.1227<br>NS	<0.0001<br>****	0.2877<br>NS	0.0019<br>**	0.9668<br>NS	0.0003<br>***
**6e**	Telomere length	**a:**control<br>**b:**cont+MitoQ<br>**c:**rotenone<br>**d:**rote+MitoQ	0.8912<br>NS	0.0067<br>**	0.9901<br>NS	0.0731<br>NS	0.7751<br>NS	0.0056<br>**
**6f**	MitoSox Red	**a:**control<br>**b:**cont+SS-31<br>**c:**rotenone<br>**d:**rote+SS-31	0.0939<br>NS	<0.0001<br>****	0.92<br>NS	0.1974<br>NS	0.4089<br>NS	0.0004<br>***
**6g**	Telomere length	**a:**control<br>**b:**cont+SS-31<br>**c:**rotenone<br>**d:**rote+SS-31	0.2348<br>NS	0.0004<br>***	0.5558<br>NS	0.0431<br>*	0.9611<br>NS	0.0214<br>*
**6i**	Telomere length	**a:**cont>cont<br>**b:**rote>cont<br>**c:**cont>rote<br>**d:**rote>rote	0.0467<br>*	>0.9999<br>NS	0.0007<br>***	0.0705<br>NS	0.6674<br>NS	0.0017<br>**

Supplementary Table 7. Statistical analysis of data presented in Figure 7.

Fig.	Parameter	Groups	a,b	a,c	a,d	b,c	b,d	c,d
**7b**	MitoSox Red	**a:**young **b:**aged **c:**aged+BGP-15	<0.0001 ****	0.0081 **	n/a	0.0268 *	n/a	n/a
**7c**	TMRM	**a:**young **b:**young+BGP-15 **c:**aged **d:**aged+BGP-15	0.8688 NS	0.0144 *	0.4206 NS	0.0476 *	0.9801 NS	0.0062 **
**7d**	Telomere length	**a:**young **b:**aged	0.0011 **	n/a	n/a	n/a	n/a	n/a
**7e**	Telomere length	**a:**nulliparous **b:**multiparous	0.0304 *	n/a	n/a	n/a	n/a	n/a
**7f**	Telomere length	**a:**young **b:**young+BGP-15 **c:**aged **d:**aged+BGP-15	>0.9999 NS	0.9986 NS	0.9705 NS	0.9979 NS	0.9669 NS	0.9971 NS
**7g**	Telomere length	**a:**young **b:**aged **c:**aged+BGP-15	<0.0001 ****	0.0430 *	n/a	0.0305 *	n/a	n/a
**7h**	Telomere length	**a:**young **b:**aged **c:**aged+BGP-15	0.0364 *	0.8798 NS	n/a	0.0095 **	n/a	n/a
**7j**	MitoSox Red	**a:**young **b:**aged **c:**aged+metformin **d:**aged+MitoQ	0.0330 *	0.3378 NS	0.0873 NS	<0.0001 ****	<0.0001 ****	0.9874 NS
**7k**	Telomere length	**a:**young **b:**aged **c:**aged+metformin **d:**aged+MitoQ	0.0014 **	0.0019 **	0.8236 NS	0.9994 NS	0.0412 *	0.0418 *

Supplementary Table 8. Statistical analysis of data presented in Figure 8.

Fig.	Parameter	Groups	a,b	a,c	a,d	b,c	b,d	c,d
**8b**	MitoSox Red	**a:**lean **b:**lean+BGP-15 **c:**obese **d:**obese+BGP-15	0.6382 NS	<0.0001 ****	0.9961 NS	<0.0001 ****	0.3683 NS	<0.0001 ****
**8c**	TMRM	**a:**lean **b:**obese **c:**obese+BGP-15	0.0005 ***	0.9970 NS	n/a	0.0009 ***	n/a	n/a
**8d**	Telomere length	**a:**lean **b:**lean+BGP-15 **c:**obese **d:**obese+BGP-15	0.8019 NS	0.0218 *	>0.9999 NS	0.0032 **	0.8185 NS	0.0420 *
**8e**	MitoSox Red	**a:**lean **b:**lean+SS-31 **c:**obese **d:**obese+SS-31	0.9490 NS	<0.0001 ****	0.0431 *	0.0037 **	0.0053 **	<0.0001 ****
**8f**	Telomere length	**a:**lean **b:**lean+SS-31 **c:**obese **d:**obese+SS-31	0.1460 NS	0.0006 ***	0.9794 NS	0.2679 NS	0.2565 NS	0.0013 **

Supplementary Table 9. Statistical analysis of data presented in Figure 9.

Fig.	Parameter	Groups	a,b	a,c	a,d	b,c	b,d	c,d
**9b**	MitoSox Red	**a:**lean **b:**lean+BGP-15 **c:**obese **d:**obese+BGP-15	0.9675 NS	0.0017 **	0.9982 NS	0.0277 *	0.9998 NS	0.0140 *
**9c**	TMRM (8-cell)	**a:**lean **b:**lean+BGP-15 **c:**obese **d:**obese+BGP-15	0.5658 NS	0.0002 ***	0.0051 **	0.0002 ***	0.4814 NS	<0.0001 ****
**9c**	TMRM (morula)	**a:**lean **b:**lean+BGP-15 **c:**obese **d:**obese+BGP-15	0.0455 *	0.0029 **	0.9874 NS	<0.0001 ****	0.4492 NS	0.0015 **
**9d**	Telomere length	**a:**lean **b:**lean+BGP-15 **c:**obese **d:**obese+BGP-15	0.0353 *	0.0038 **	0.6737 NS	0.9522 NS	0.0786 NS	0.3676 NS
**9e**	Telomere length	**a:**lean **b:**lean+BGP-15 **c:**obese **d:**obese+BGP-15	0.8959 NS	0.6326 NS	0.9829 NS	0.2123 NS	0.6147 NS	0.7763 NS
**9f**	Telomere length	**a:**lean **b:**lean+BGP-15 **c:**obese **d:**obese+BGP-15	0.2504 NS	0.0007 ***	0.8896 NS	0.1727 NS	0.8557 NS	0.0444 *
**9g**	Telomere length	**a:**lean **b:**obese **c:**obese+BGP-15	0.0166 *	0.1750 NS	n/a	0.0003 ***	n/a	n/a
**9h**	MitoSox Red	**a:**lean **b:**obese **c:**obese+Metformin **d:**obese+MitoQ	0.0004 ***	0.9549 NS	0.8725 NS	0.0079 **	0.0087 **	>0.9999 NS
**9i**	Telomere length	**a:**lean **b:**obese **c:**obese+Metformin **d:**obese+MitoQ	0.0042 **	0.9034 NS	0.8080 NS	0.0016 **	0.0007 ***	0.9978 NS
**9j**	Telomere length (liver)	**a:**lean **b:**obese **c:**obese+BGP-15	<0.0001 ****	0.0025 **	n/a	<0.0001 ****	n/a	n/a
**9j**	Telomere length (kidney)	**a:**lean **b:**obese **c:**obese+BGP-15	0.0223 *	0.1568 NS	n/a	0.7137 NS	n/a	n/a
**9j**	Telomere length (heart)	**a:**lean **b:**obese **c:**obese+BGP-15	<0.0001 ****	0.0018 **	n/a	0.0062 88	n/a	n/a

Supplementary Table 10. Statistical analysis of data presented in Supplementary Figure S2.

Fig.	Parameter	Groups	a,b	a,c	a,d	a,e; b,e; c,e; d,e	b,c	b,d	c,d
**S2a**	Telo/cent signal	**a:**MII **b:**2-cell **c:**4-cell	<0.0001 ****	<0.0001 ****	n/a	n/a	0.0542 NS	n/a	n/a
**S2b**	Telomere length	**a:**MII **b:**2C **c:**4C **d:**8C **e:**Blast	0.041 *	0.024 *	0.013 *	<0.0001 ****	0.789 NS	0.610 NS	0.812 NS
**S2c**	Telomere length	**a:**MII **b:**2C **c:**4C **d:**8C **e:**Blast	0.740 NS	0.686 NS	0.371 NS	<0.0001 ****	0.929 NS	0.478 NS	0.532 NS
**S2d**	Telomere length	**a:**MII **b:**2C **c:**4C **d:**8C **e:**Blast	0.796 NS	0.460 NS	0.381 NS	<0.0001 ****	0.296 NS	0.230 NS	0.904 NS
**S2e**	Telomere length	**a:**MII **b:**2C **c:**4C **d:**8C **e:**Blast	0.202 NS	0.076 NS	0.001 **	<0.0001 ****	0.607 NS	0.028 *	0.096 NS
**S2f**	TTAGGG intensity	**a:**HCT116 **b:**HeLa **c:**U-2 OS	0.0067 **	<0.0001 ****	n/a	n/a	<0.0001 ****	n/a	n/a

Supplementary Table 11. Statistical analysis of data presented in Supplementary Figure S4.

Fig.	Parameter	Groups	a,b	a,c	a,d	b,c	b,d	c,d
**S4a**	Telomere length	**a:**5% fresh **b:**5% vit **c:**20% fresh **d:**20% vit	0.9964 NS	0.0196 *	0.1352 NS	0.0799 NS	0.0353 *	0.9992 NS
**S4c**	% implantations	**a:**5% **b:**20%	0.1680 NS	n/a	n/a	n/a	n/a	n/a
**S4d**	Implantation outcome	**a:**5% **b:**20%	0.4089 NS	n/a	n/a	n/a	n/a	n/a
**S4e**	Fetal rate	**a:**5% **b:**20%	0.0142 *	n/a	n/a	n/a	n/a	n/a
**S4f**	Fetal number	**a:**5% **b:**20%	0.0141 *	n/a	n/a	n/a	n/a	n/a

Supplementary Table 12. Statistical analysis of data presented in Supplementary Figure S6.

Fig.	Parameter	Groups	a,b	a,c	b,d	c,d	e,f
**S6b**	Pronuclei size	**a:**5% mat<br>**b:**5% pat<br>**c:**20% mat<br>**d:**20% pat	0.0032<br>**	0.1179<br>NS	0.0654<br>NS	<0.0001<br>****	n/a
**S6c**	5mC mean intensity	**a:**5% mat<br>**b:**5% pat<br>**c:**20% mat<br>**d:**20% pat<br>**e:**5% ratio<br>**f:**20% ratio	0.0017<br>**	0.5263<br>NS	0.0344<br>*	0.0422<br>*	0.2283<br>NS
**S6d**	5hmC mean intensity	**a:**5% mat<br>**b:**5% pat<br>**c:**20% mat<br>**d:**20% pat<br>**e:**5% ratio<br>**f:**20% ratio	0.0011<br>**	0.0619<br>NS	0.2528<br>NS	0.0621<br>NS	0.0292<br>*

Supplementary Table 13. Statistical analysis of data presented in Supplementary Figure S7.

Fig.	Parameter	Groups	a,b
**S7b**	Fetal number	**a:**control<br>**b:**rotenone	0.9072<br>NS
**S7c**	Fetal weight	**a:**control<br>**b:**rotenone	0.7795<br>NS
**S7d**	Fetal sex	**a:**control<br>**b:**rotenone	0.4128<br>NS
**S7e**	Telomere length	**a:**control<br>**b:**rotenone	0.7060<br>NS
**S7f**	Telomere length	**a:**control<br>**b:**rotenone	0.0027<br>**

Supplementary Table 14. Statistical analysis of data presented in Supplementary Figure S8.

Fig.	Parameter	Groups	a,b	a,c	b,d	c,d	e,f
**S8b**	Pronuclei size	**a:**control mat<br>**b:**control pat<br>**c:**rotenone mat<br>**d:**rotenone pat	<0.0001<br>****	0.8615<br>NS	0.6346<br>NS	0.0170<br>*	n/a
**S8c**	5mC mean intensity	**a:**control mat<br>**b:**control pat<br>**c:**rotenone mat<br>**d:**rotenone pat<br>**e:**control ratio<br>**f:**rotenone ratio	<0.0001<br>****	0.6438<br>NS	0.2227<br>NS	0.0249<br>*	0.0441<br>*
**S8d**	5hmC mean intensity	**a:**control mat<br>**b** control pat<br>**c:**rotenone mat<br>**d:**rotenone pat<br>**e:**control ratio<br>**f:**rotenone ratio	<0.0001<br>****	0.0283<br>*	0.4195<br>NS	0.0003<br>***	0.0038<br>**

Supplementary Table 15. Statistical analysis of data presented in Supplementary Figure S9.

Fig.	Parameter	Groups	a,b	a,c	a,d	b,c	b,d	c,d
**S9a**	Telomere length	**a:**control fresh<br>**b:**control vit<br>**c:**rotenone fresh<br>**d:**rotenone vit	0.9816<br>NS	0.0142<br>*	0.0703<br>NS	0.1877<br>NS	0.0474<br>*	0.8755<br>NS
**S9c**	% implantations	**a:**control<br>**b:**rotenone	0.7723<br>NS	n/a	n/a	n/a	n/a	n/a
**S9d**	Implantation outcome	**a:**control<br>**b:**rotenone	0.9593<br>NS	n/a	n/a	n/a	n/a	n/a
**S9e**	Fetal rate	**a:**control<br>**b:**rotenone	0.7124<br>NS	n/a	n/a	n/a	n/a	n/a
**S9f**	Fetal number	**a:**control<br>**b:**rotenone	0.7139<br>NS	n/a	n/a	n/a	n/a	n/a
**S9g**	Fetal weight	**a:**control<br>**b:**rotenone	0.8835<br>NS	n/a	n/a	n/a	n/a	n/a
**S9h**	Fetal sex	**a:**control<br>**b:**rotenone	0.6185<br>NS	n/a	n/a	n/a	n/a	n/a
**S9i**	Telomere length	**a:**control<br>**b:**rotenone	0.3654<br>NS	n/a	n/a	n/a	n/a	n/a
**S9j**	Telomere length	**a:**control<br>**b:**rotenone	0.7518<br>NS	n/a	n/a	n/a	n/a	n/a
**S9k**	Telomere length	**a:**control<br>**b:**rotenone	0.9778<br>NS	n/a	n/a	n/a	n/a	n/a
**S9l**	Telomere length	**a:**control<br>**b:**rotenone	0.1309<br>NS	n/a	n/a	n/a	n/a	n/a

Supplementary Table 16. Statistical analysis of data presented in Supplementary Figure S10.

Fig.	Parameter	Groups	a,b	a,c	a,d	b,c	b,d	c,d
**S10a**	MitoSox Red	**a:**control<br>**b:**rotenone	<0.0001<br>****	n/a	n/a	n/a	n/a	n/a
**S10c**	%2C	**a:**cont>cont<br>**b:**rote>cont<br>**c:**cont>rote<br>**d:**rote>rote	0.9302<br>NS	0.9961<br>NS	0.9414<br>NS	0.8436<br>NS	>0.9999<br>NS	0.8608<br>NS
**S10d**	% blast	**a:**cont>cont<br>**b:**rote>cont<br>**c:**cont>rote<br>**d:**rote>rote	0.9878<br>NS	0.9860<br>NS	0.2935<br>NS	>0.9999<br>NS	0.4540<br>NS	0.4627<br>NS
**S10e**	% hatching blast	**a:**cont>cont<br>**b:**rote>cont<br>**c:**cont>rote<br>**d:**rote>rote	0.9534<br>NS	0.9833<br>NS	0.9657<br>NS	0.9986<br>NS	>0.9999<br>NS	0.9996<br>NS

Supplementary Table 17. Statistical analysis of data presented in Supplementary Figure S11.

Fig.	Parameter	Groups	a,b	a,c	a,d	a,e	b,c	b,d	b,f	c,d	c,e	d,f	e,f
**S11a**	8-oxodG	**a:**young<br>**b:**young+B<br>**c:**aged<br>**d:**aged+B	0.1266<br>NS	0.0367<br>*	0.0031<br>**	n/a	0.8379<br>NS	0.347<br>NS	n/a	0.8925<br>NS	n/a	n/a	n/a
**S11c**	Pronuclei size	**a:**young mat<br>**b:**young pat<br>**c:**aged mat<br>**d:**aged pat<br>**e:**aged+B mat<br>**f:** aged+B pat	<0.0001<br>****	0.4102<br>NS	n/a	0.8549<br>NS	n/a	0.997<br>NS	0.1438<br>NS	0.0043<br>**	0.6972<br>NS	0.069<br>NS	<0.0001<br>****
**S11d**	5mC mean intensity	**a:**young mat<br>**b:**young pat<br>**c:**aged mat<br>**d:**aged pat<br>**e:**aged+B mat<br>**f:** aged+B pat	0.0121<br>*	<0.0001<br>****	n/a	0.9274<br>NS	n/a	0.155<br>NS	0.9537<br>NS	0.3575<br>NS	<0.0001<br>****	0.198<br>NS	0.0015<br>**
**S11e**	5mC mean intensity	**a:**young<br>**b:**aged<br>**c:**aged+B	0.1797<br>NS	0.9141<br>NS	n/a	n/a	0.0459<br>*	n/a	n/a	n/a	n/a	n/a	n/a
**S11f**	5hmC mean intensity	**a:**young mat<br>**b:**young pat<br>**c:**aged mat<br>**d:**aged pat<br>**e:**aged+B mat<br>**f:** aged+B pat	0.0001<br>***	0.9861<br>NS	n/a	0.0013<br>**	n/a	0.253<br>NS	0.0602<br>NS	<0.0001<br>****	0.0007<br>****	0.645<br>NS	0.0038<br>**
**S11g**	5hmC mean intensity	**a:**young<br>**b:**aged<br>**c:**aged+B	0.3381<br>NS	0.1704<br>NS	n/a	n/a	0.0018<br>**	n/a	n/a	n/a	n/a	n/a	n/a

Supplementary Table 18. Statistical analysis of data presented in Supplementary Figure S12.

Fig.	Parameter	Groups	a,b	a,c	a,d	a,e	b,c	b,d	b,f	c,d	c,e	d,f	e,f
**S12a**	TMRM	**a:**lean<br>**b:**obese<br>**c:**obese+B	0.0003<br>***	0.021<br>*	n/a	n/a	0.1656<br>NS	n/a	n/a	n/a	n/a	n/a	n/a
**S12b**	8-oxodG	**a:**lean<br>**b:**lean+B<br>**c:**obese<br>**d:**obese+B	0.2503<br>NS	0.8433<br>NS	0.882<br>NS	n/a	0.6509<br>NS	0.0912<br>NS	n/a	0.4548<br>NS	n/a	n/a	n/a
**S12d**	Pronuclei size	**a:**lean mat<br>**b:**lean pat<br>**c:**ob mat<br>**d:**ob pat<br>**e:**ob+B mat<br>**f:**ob+B pat	<0.0001<br>****	0.1852<br>NS	n/a	0.8305<br>NS	n/a	<0.0001<br>****	0.9955<br>NS	0.0002<br>***	0.4656<br>NS	0.0001<br>***	<0.0001<br>****
**S12e**	5mC mean intensity	**a:**lean mat<br>**b:**lean pat<br>**c:**ob mat<br>**d:**ob pat<br>**e:**ob+B mat<br>**f:**ob+B pat	<0.0001<br>****	0.03662<br>*	n/a	0.3798<br>NS	n/a	0.0064<br>**	0.561<br>NS	0.3842<br>NS	0.3871<br>NS	0.0803<br>NS	0.0013<br>**
**S12f**	5mC mean intensity	**a:**lean<br>**b:**obese<br>**c:**obese+B	0.0831<br>NS	0.8006<br>NS	n/a	n/a	0.0290<br>*	n/a	n/a	n/a	n/a	n/a	n/a
**S12g**	5hmC mean intensity	**a:**lean mat<br>**b:**lean pat<br>**c:**ob mat<br>**d:**ob pat<br>**e:**ob+B mat<br>**f:**ob+B pat	<0.0001<br>****	0.2634<br>NS	n/a	0.1268<br>NS	n/a	0.0388<br>*	0.842<br>NS	0.0013<br>**	0.995<br>NS	0.1429<br>NS	<0.0001<br>****
**S12h**	5hmC mean intensity	**a:**lean<br>**b:**obese<br>**c:**obese+B	<0.0001<br>****	0.1089<br>NS	n/a	n/a	0.2113<br>NS	n/a	n/a	n/a	n/a	n/a	n/a
