# Supplementary material for: Telomere length in offspring is determined by mitochondrial-nuclear communication at fertilization
Source: Nat Commun. 2025 Mar 14;16:2527. doi: 10.1038/s41467-025-57794-7 (PMC11909127; doi:10.1038/s41467-025-57794-7)
Supplement: Supplementary file 2 — Reporting Summary [file 41467_2025_57794_MOESM2_ESM.pdf]

Corresponding author(s): Rebecca Robker
Last updated by author(s): Feb 3, 2025

Reporting Summary

Nature Portfolio wishes to improve the reproducibility of the work that we publish. This form provides structure for consistency and transparency in reporting. For further information on Nature Portfolio policies, see our Editorial Policies and the Editorial Policy Checklist.

Statistics

For all statistical analyses, confirm that the following items are present in the figure legend, table legend, main text, or Methods section.

n/a	Confirmed	
☐	☒	The exact sample size (*n*) for each experimental group/condition, given as a discrete number and unit of measurement
☐	☒	A statement on whether measurements were taken from distinct samples or whether the same sample was measured repeatedly
☐	☒	The statistical test(s) used AND whether they are one- or two-sided<br>*Only common tests should be described solely by name; describe more complex techniques in the Methods section.*
☒	☐	A description of all covariates tested
☐	☒	A description of any assumptions or corrections, such as tests of normality and adjustment for multiple comparisons
☐	☒	A full description of the statistical parameters including central tendency (e.g. means) or other basic estimates (e.g. regression coefficient) AND variation (e.g. standard deviation) or associated estimates of uncertainty (e.g. confidence intervals)
☐	☒	For null hypothesis testing, the test statistic (e.g. *F*, *t*, *r*) with confidence intervals, effect sizes, degrees of freedom and *P* value noted<br>*Give P values as exact values whenever suitable.*
☒	☐	For Bayesian analysis, information on the choice of priors and Markov chain Monte Carlo settings
☒	☐	For hierarchical and complex designs, identification of the appropriate level for tests and full reporting of outcomes
☒	☐	Estimates of effect sizes (e.g. Cohen's *d*, Pearson's *r*), indicating how they were calculated

Our web collection on statistics for biologists contains articles on many of the points above.

Software and code

Policy information about availability of computer code

Data collection	No software was used.
Data analysis	GraphPad Prism 10.1.0 (GraphPad Software Inc., La Jolla, CA)<br>Image J Fiji version 1.0<br>ZEN 3.4 software (Zeiss)<br>Cellprofiler v4.2.1<br>SPSS Statistics 29.0.1.0 (IBM, Armonk, NY)

For manuscripts utilizing custom algorithms or software that are central to the research but not yet described in published literature, software must be made available to editors and reviewers. We strongly encourage code deposition in a community repository (e.g. GitHub). See the Nature Portfolio guidelines for submitting code & software for further information.

Data

Policy information about availability of data

All manuscripts must include a data availability statement. This statement should provide the following information, where applicable:

- Accession codes, unique identifiers, or web links for publicly available datasets
- A description of any restrictions on data availability
- For clinical datasets or third party data, please ensure that the statement adheres to our policy

Data and materials availability: All data are available in the main text or the supplementary materials.

Research involving human participants, their data, or biological material

Policy information about studies with human participants or human data. See also policy information about sex, gender (identity/presentation), and sexual orientation and race, ethnicity and racism.

Reporting on sex and gender	There were no human research participants.
Reporting on race, ethnicity, or other socially relevant groupings	There were no human research participants.
Population characteristics	There were no human research participants.
Recruitment	There were no human research participants.
Ethics oversight	There were no human research participants.

Note that full information on the approval of the study protocol must also be provided in the manuscript.

Field-specific reporting

Please select the one below that is the best fit for your research. If you are not sure, read the appropriate sections before making your selection.

☒ Life sciences ☐ Behavioural & social sciences ☐ Ecological, evolutionary & environmental sciences

For a reference copy of the document with all sections, see nature.com/documents/nr-reporting-summary-flat.pdf

Life sciences study design

All studies must disclose on these points even when the disclosure is negative.

Sample size	Samples sizes were based on our previous study (Wu et.al Development 2015 142:681) using the same strains of mice and similar physiological outcomes, primarily embryo development in this manuscript.
Data exclusions	No data were excluded.
Replication	Mouse physiological measures (ie IVF/ embryo development) and live cell assays (ie mitochondrial TMRM and MSR staining) were conducted in manageable cohorts and the data from each replicate pooled. Tissues collected in cohorts underwent biochemical assays (ie immunocytochemistry, qPCR) simultaneously. Each animal or pool of cells from multiple animals was considered an independent biological replicate. Only biological replicates were statistically analyzed and reported in the manuscript. All attempts at replication were successful.
Randomization	Mice were always randomly allocated to the different treatment groups, for instance rotenone diet or control diet. Mice that were obese, old, or exposed to rotenone diet were randomly assigned to receive drug or vehicle.
Blinding	Investigators were blinded to treatment group during oocyte collections and were not generally aware of treatment groups during tissue processing and biochemical assessments that generate quantitative data.

Reporting for specific materials, systems and methods

We require information from authors about some types of materials, experimental systems and methods used in many studies. Here, indicate whether each material, system or method listed is relevant to your study. If you are not sure if a list item applies to your research, read the appropriate section before selecting a response.

Materials & experimental systems

n/a	Involved in the study
☐	☒ Antibodies
☐	☒ Eukaryotic cell lines
☒	☐ Palaeontology and archaeology
☐	☒ Animals and other organisms
☒	☐ Clinical data
☒	☐ Dual use research of concern
☒	☐ Plants

Methods

n/a	Involved in the study
☒	☐ ChIP-seq
☒	☐ Flow cytometry
☒	☐ MRI-based neuroimaging

Antibodies

Antibodies used

All antibodies used in the study are commercially available. Antibody catalog numbers , suppliers and the dilutions at which they were used are detailed in the Methods.
Antibodies (source, dilution, supplier, cat, clone, lot):
8-oxo: mouse, 1:10, Nikken SEIL Co. Ltd, #MOG-100P, clone N45.1, Lot. 013 MOG-100P
5mC: mouse, 1:200, Biorad, #MCA2201, clone 33D3, Lot. 170919
5hmC: rabbit, 1:600, Active Motif, #39769, Lot. 01218002

Validation

Antibodies were each validated by the commercial source. No in-house or previously unpublished antibodies were used in these studies. The catalog numbers and company are provided in the Methods.
In each case the normal localization/ cell specificity is well understood and only relative amounts of staining were measured in these studies.
Negative controls were included in each experiment:
8-oxo: performed both a 'no primary' negative control and a 'DNAse-treated' negative control, both returned no staining
5mC: performed a 'no primary' negative control
5hmC: performed a 'no primary' negative control

Eukaryotic cell lines

Policy information about cell lines and Sex and Gender in Research

Cell line source(s)

HCT116, HeLa and U-2-OS cancer cell lines. In house or from CellBank Australia. In house or from CellBank Australia.

Authentication

All cell lines were verified by STR profiling through CellBank Australia.

Mycoplasma contamination

All cell lines were tested and clear for Mycoplasma be CellBank Australia.

Commonly misidentified lines
(See ICLAC register)

N/A

Animals and other research organisms

Policy information about studies involving animals; ARRIVE guidelines recommended for reporting animal research, and Sex and Gender in Research

Laboratory animals

Animals (source, substrain, genetic background, species, age):
CBA: University of Adelaide's Laboratory Animal Services, mouse, CBA/CaHArc, females 6-7 weeks of age, males 6-8 weeks of age.
C57: Animal Resources Centre Western Australia, mouse, C57BL6/Arc, females 6-7 weeks of age, males 6-8 weeks of age.
CBAF1: University of Adelaide's Laboratory Animal Services, mouse, CBA/CaHArc x C57BL6/Arc, females 6-7 weeks of age, males 6-8 weeks of age.
Blobby: University of Adelaide colony, mouse, C57BL/6JSfdAnu-Alms1bbb/Apb, females 6-7 weeks of age, 4-5months of age (obesity), 3-4 months of age (young controls) or 12 months of age (aged), males 6-8 weeks of age.

All mice were maintained in 12h/12h light/dark conditions with ambient temperature of 22Ċ and 40-60% humidity. Mice were given water and rodent chow (Teklad Global 19% Protein Extruded Rodent Diet, #2019) ad libitum.

Mice of the C57BL/6JSfdAnu-Alms1bbb/Apb ('Blobby') strain were used to generate obese and aged mice and control litter mates.
The C57BL/6JSfdAnu-Alms1bbb/Apb mouse strain was originally sourced from the Australian Phenome Bank (APB ID 31; MGI:3611799) and a colony established at the University of Adelaide. Females were used and they were 4-5 months old or 12 months old as indicated in each experiment.
Female mice (7-10 weeks old) of the CBAxC57 F1 (CBA.F1) strain were used in the rotenone-diet experiments and these were sourced from a breeding colony managed by Laboratory Animal Services at the University of Adelaide.
Males of each strain were only used as sperm donors to generate embryos, males of the C57BL/6JSfdAnu-Alms1bbb/Apb strain were wild-type.

Wild animals	No wild animals were used in this study.
Reporting on sex	Female biology is the focus of this study and the use of female mice is certainly stated throughout the Results, Figure Legends and Methods.
Field-collected samples	No field-collected animals or samples were used in this study.
Ethics oversight	All animal experiments were approved by the University of Adelaide's Animal Ethics Committee and conducted in accordance with the Australian Code of Practice for the Care and Use of Animals for Scientific Purposes.

Note that full information on the approval of the study protocol must also be provided in the manuscript.
